# Linkage analysis and association analysis in the presence of linkage using age at onset of COGA alcoholism data

**DOI:** 10.1186/1471-2156-6-S1-S31

**Published:** 2005-12-30

**Authors:** Xiaoyun Zhong, Heping Zhang

**Affiliations:** 1 Yale University School of Medicine, New Haven, Connecticut, USA

## Abstract

Complex disease mapping usually involves a combination of linkage and association techniques. Linkage analysis can scan the entire genome in a few hundred tests. Association tests may involve an even greater number of tests. However, association tests can localize the susceptibility genes more accurately. Using a recently developed combined linkage and association strategy, we analyzed a subset of the Collaborative Study on the Genetics of Alcoholism (COGA) data for the Genetic Analysis Workshop 14 (GAW14). In this analysis, we first employed linkage analysis based on frailty models that take into account age of onset information to establish which regions along the chromosome are likely to harbor disease susceptibility genes for alcohol dependence. Second, we used an association analysis by exploiting linkage disequilibrium to narrow down the peak regions. We also compare the methods with mean identity-by-descent tests and transmission/disequilibrium tests that do not use age of onset information.

## Background

The Collaborative Study on the Genetics of Alcoholism (COGA) is a large, multi-site genetic study to identify susceptibility genes for alcohol dependence and related phenotypes [1]. The COGA data have been analyzed using nonparametric sib-pair methods with the two-point linkage program and multipoint linkage program for affected sib pairs [2]. Linkage signals were revealed on chromosomes 1, 2, 4, and 7.

Age of onset data are often collected in studies designed to map a complex disease. If age at onset is genetically mediated, it may carry useful linkage information. Genetic analysis that incorporates variable age of onset may improve the ability to map genes for complex diseases. In this report, we analyzed the COGA data using genetic methods based on additive genetic gamma frailty models to account for age of onset or covariate information [3,4].

## Methods

Consider a sibship with *n *sibs. Let *T*_*j *_be the random variable of age at disease onset for the *j*^th ^sib. Let (*t*_*j*_,*δ*_*j*_) be the observed data where *t*_*j *_is the observed age at onset if *δ*_*j *_= 1, and age at censoring if *δ*_*j *_= 0. Consider a marker locus *d *in the test chromosomal region. We assume that the hazard function of developing disease for the *j*^th ^individuals at age *t*_*j *_is modelled by the proportional hazards model with random effect *Z*_*j*_,

λ_*j*_(*t*_*j*_|*Z*_*j*_) = λ_0_(*t*_*j*_)exp(*X*_*j*_β)*Z*_*j*_, for *j *= 1, 2, ..., *n*,

where λ_0_(*t*) is the unspecified baseline hazard function, and *X*_*j *_is a vector of observed covariates for the *j*^th ^sib and *β *is a vector of regression parameters associated with the covariates. *Z*_*j *_is the unobserved genetic frailty. The genetic frailty is defined as the following



where *V*_*d *_= (*v*_1_, *v*_2_, ..., *v*_2*n *- 1_, *v*_2*n*_) is the inheritance vector [5] of a sibship at locus *d*, *v*_2*j *- 1 _= 1 or 2, and *v*_2*j *_= 3 or 4 to indicate the origins of the inherited alleles for *j *= 1, 2, ..., n. *U*_*d*1 _and *U*_*d*2 _represent the genetic frailties due to part of the genome on the two chromosomes of the father at locus *d*. *U*_*d*3 _and *U*_*d*4 _represent the same, though for the mother. The random frailty term, *U*_*p*_, takes into account possible genetic contributions to shared familial effects. Gamma distributions were used to model the frailties and retrospective likelihood ratio tests were constructed for linkage analysis [3]. After linkage evidence is established by the linkage analysis [3], we used an association test in the presence of linkage as proposed by Zhong and Li [4] that examines the putative association between the disease and the testing allele at a candidate chromosomal locus in the linked region. Because we use age-at-onset as the outcome, the association test is based on the proportional hazards model.

The dataset includes a total of 143 nuclear and multigenerational families with 1,614 individuals. There are two kinds of diagnostic definitions of alcohol dependence, labelled in the data set as ALDX1 and ALDX2. ALDX1 alcohol-dependent subjects were defined as those individuals who met both the DSM-III-R (Diagnostic and Statistical Manual of the American Psychiatric Association-Revised) criteria for alcohol dependence and the Feighner criteria for alcoholism. ALDX2 alcohol-dependent subjects were defined as those who met the DSM-IV criteria. Both ALDX1 and ALDX2 alcohol dependence phenotypes are coded in four levels: pure unaffected, never drank, unaffected with some symptoms, and affected. We combined the first three codings as unaffected. We extracted genotyped affected sib pairs with their parents from each pedigree to ensure independence between nuclear families. This yielded 142 affected sib pairs with their parents for a total of 568 individuals for the ALDX1 phenotype for alcoholism. For the ALDX2 phenotype, this yielded 117 affected sib pairs with their parents for a total of 468 individuals. We utilized MAPMAKER/SIBS linkage program [6] to estimate the full multipoint probability of each pair of selected sibs sharing 0, 1, or 2 alleles identical by descent (IBD) on each chromosome. We obtained the Kaplan-Meier curves as the approximation to the baseline functions using the available age-of-onset data from all of the founders in the full dataset (287 for ALDX1 and 289 for ALDX2). The founders are individuals who do not have parents in pedigree and are considered as random subjects from the general population. The Kaplan-Meier survival curves for females and males (figures not shown here) indicate strong evidence of sex differences in ages of onset distributions. Alcohol dependence is more common in males than females. The Kaplan-Meier survival curves for smokers and nonsmokers (figures not shown here) also indicate distributional differences between smokers and nonsmokers.

For each of the two disease classifications, ALDX1 and ALDX2, we first performed linkage analysis over the whole genome, excluding the sex chromosome, using the methods of Li and Zhong [3], adjusting sex and smoking status as the covariates as well as the mean IBD test to analyze the microsatellite markers on each chromosome for linkage. The mean IBD test determines whether affected sib pairs share alleles at a specific marker more than the Mendelian expectation under no linkage. After the linkage evidence (*p*-value < 0.01) to some candidate genes is established, we further applied the association method [4] as well as the transmission/disequilibrium tests [7] to single-nucleotide polymorphism (SNP) markers within the peak regions.

## Results

A preliminary multipoint genome scan using mean IBD tests (figures not shown here) indicated some evidence of linkage to ALDX1 in the regions around 145 cM of chromosome 7 (*p*-value of 0.006), 161 cM of chromosome 6 (*p*-value of 0.008), and 157 cM of chromosome 12 (*p*-value of 0.016). Evidence of linkage to ALDX2 was found in the regions around 128 cM of chromosome 7 (*p*-value of 0.0086), 14 cM of chromosome 8 (*p*-value of 0.0196), 169 cM of chromosome 6 (*p*-value of 0.021), 5 cM of chromosome 2 (*p*-value of 0.041), and 148 cM of chromosome 2 (*p*-value of 0.049).

**Figure 1:**
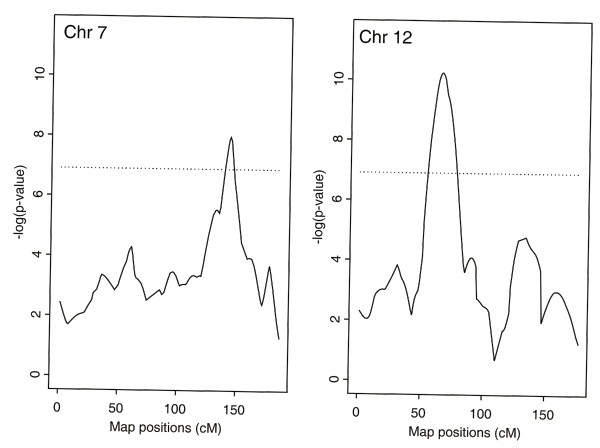
**The multipoint linkage scans over chromosome 7 for ALDX1 and chromosome 12 for ALDX2, adjusting gender and smoking status as covariates. **The dashed horizontal lines refer to the negative of the natural logarithm of 0.001 (i.e., -log(0.001)) corresponding to the significance level of 0.001.

Table 1 lists the names and the map positions in centimorgans of the markers near those peaks with *p*-values less than 0.01 for genome scans using frailty models for linkage incorporating gender as a covariate or gender and smoking status as covariates for both ALDX1 and ALDX2. Frailty models identified the regions with evidence of linkage by the mean IBD tests, but with stronger signals, and also revealed some new regions with significant evidence of linkage. Adjusting for gender and smoking status as covariates, the strongest evidence of linkage to ALDX1 was achieved on a region on chromosome 7 (Table 1). Some evidence of linkage to ALDX2 was also found close to that region. The strongest evidence of linkage to ALDX2 was obtained on a region on chromosome 12. The same region was also found with some evidence of linkage to ALDX1. Figure 1 displays the multipoint linkage scans over chromosome 7 for ALDX1 and chromosome 12 for ALDX2, adjusting gender and smoking status as covariates.

For the association tests in the presence of linkage, we present here only the SNPs within a 10-cM vicinity of the two linkage peaks with smallest *p*-values under each of disease criteria. For ALDX2, evidence of association at a 0.001 significant level using frailty models adjusting gender and smoking status as covariates was found for SNPs *rs273954 *and *rs700273 *on chromosome 7, and *rs710411 *on chromosome 9 and ALDX1, and between *rs1978161*, *rs1565933*, *rs1867299*, *rs2279400*, *rs1848125*, *rs1224438*, *rs1495042*, *rs965125*, and *rs1444588 *on chromosome 12, and *rs1039559 *on chromosome 4. As a comparison, for ALDX1, evidence of association at a 0.05 level using transmission/disequilibrium test on trio (parents and one affected sib) data was found between *rs273954 *and *rs727714 *on chromosome 7; for ALDX2, evidence was found for *rs12142 *and *rs951149 *on chromosome 4, and *rs1705748*, *rs1705772*, *rs1843910*, *rs1846629*, and *rs1820545 *on chromosome 12.

## Discussion

Although the markers identified under the two criteria, ALDX1 and ALDX2, are not entirely the same, several common regions were identified. The similarity of the regions supports some common genetic bases for ALDX1 and ALDX2, whereas the differences in the identified regions underscore the importance of using different phenotypes. As studied in [3,4], the methods using frailty models have correct type I error rates. The methods using frailty models incorporate age at onset and covariate factors and can increase the power of detecting linkage evidence over the traditional methods such as mean IBD test, which does not make use of age at onset information (Table 1). It is also interesting to note that the inclusion of smoking as a covariate in the linkage analysis resulted in more candidate genes with significant linkage evidence. We should note that the *p*-values reported here are not corrected for multiple comparisons.

## Abbreviations

COGA: Collaborative Study on the Genetics of Alcoholism

GAW: Genetic Analysis Workshop

IBD: Identical by descent

SNP: Single-nucleotide polymorphism

## Authors' contributions

XZ conceived of the study, carried out the genetic studies, performed the statistical analysis and drafted the manuscript. HZ drafted the manuscript, revised it critically for important intellectual content, and gave final approval of the version to be published. All authors read and approved the final manuscript.

## Figures and Tables

**Table 1:** Table of linkage results

	Sex			Sex + Smoking		
		
Chr	Markers	Position(cM)	*P*-value	Markers	Position(cM)	*P*-value
ALDX1						
2	D2S1329	4.9	0.0098	D2S1319	4.9	0.008
2				D2S1790	114.2	0.001
2				D2S2370	184.3	0.006
2				D2S1323	251.9	0.002
3				D3S2398	216.5	0.006
4	D4S1651	110.3	0.003	D4S1651	110.3	0.003
6	GATA165G02	160.4	0.003	GATA165G02	160.4	0.003
7	D7S490	145.5	0.004	D7S490	145.5	0.0003
9				DBH.PCR2.1	165.5	0.0007
10				D10S1213	134.1	0.005
12	D12S390	67.9	0.007	D12S390	67.9	0.007
12	D12S2078	156.8	0.003	D12S2078	156.8	0.003
ALDX2						
2	D2S1328	147.6	0.002	D2S1328	147.6	0.002
3				D3S2398	216.5	0.007
4	GABRB1	51.4	0.0007	GABRB1	51.4	0.0007
5				D5S1473	34	0.003
6	D6S1007	168.6	0.009			
7	D7S1799	127.7	0.003	D7S1799	127.7	0.002
8	D8S1145	13.6	0.005	D8S1145	13.6	0.005
9				DBH.PCR2.1	165.5	0.001
12				D12S390	67.9	0.00004
13				D13S325	34.1	0.006
15				D15S816	122.6	0.006
16				D16S423	8.2	0.004
20	D20S94	80.9	0.002	D20S94	80.9	0.001

Summary of linkage markers (*P*-value < 0.01) from multipoint genome scan.
